# Hepatocellular Carcinoma Growth Is Inhibited by *Euphorbia helioscopia* L. Extract in Nude Mice Xenografts

**DOI:** 10.1155/2015/601015

**Published:** 2015-05-18

**Authors:** Junsheng Cheng, Wei Han, Zheyuan Wang, Yuan Shao, Yingzhen Wang, Yawu Zhang, Zhongxin Li, Xiaodong Xu, Youcheng Zhang

**Affiliations:** ^1^Department of General Surgery, Lanzhou University Second Hospital, Cuiyingmen 82, Chengguan District, Lanzhou 730030, China; ^2^Hepato-Biliary-Pancreatic Institute, Lanzhou University Second Hospital, Lanzhou 730030, China; ^3^Gansu Provincial-Level Key Laboratory of Digestive System Tumors, Lanzhou 730030, China; ^4^Department of General Surgery, Gansu Provincial Second Hospital, Lanzhou 730030, China

## Abstract

*Euphorbia helioscopia* L. is a traditional Chinese medicine; recently research found that its ethyl acetate extract (EAE) plays an important role on tumor cell proliferation, apoptosis, invasion, and metastasis *in vitro*. But the effect of EAE for tumor cells *in vivo* has not been reported. To explore the inhibitory effect of EAE and molecular mechanism on hepatocellular carcinoma (HCC) SMMC-7721 cells *in vivo*, we utilized the nude mouse xenograft model of HCC. Treated with EAE (50, 100, and 200 *μ*g/mL), the volume of xenograft was measured during the entire process of EAE treatment. In EAE treatment group, the volume of xenograft was significantly reduced compared with the control group (*P* < 0.05) and the protein expressions of CyclinD1, bcl-2, and MMP-9 were reduced, while those of bax, caspase-3, and nm23-H1 were increased. A significant change trend with increasing EAE concentrations has presented, compared with controls. Moreover, the ultrastructural morphology of xenografts showed significant changes, including nuclear pyknosis and chromatin condensation, We found that EAE could effectively inhibit tumor growth, induce apoptosis, and inhibit tumor invasion and metastasis *in vivo*; it is suggested that EAE is a potential candidate for as a new anticancer agent.

## 1. Introduction

Hepatocellular carcinoma (HCC), the fifth most common neoplasm worldwide [1], is one of the primary causes of cancer-related death in the world [2]. Since the 1990s, HCC has become the second leading cause of cancer death in China. Importantly, diagnosis in the advanced stages, paucity of effective therapeutic options, and high rate of tumor recurrence give rise to its high lethality [3]. Although the methods of diagnosis and treatment in liver cancer made a greatly progress, as an aggressive solid tumor, its prognosis was poor [4]. The 5-year survival is 35–45% after surgical resection [5, 6] and 47–61% after transplantation [7].

In China, surgical resection has been accepted as one of the best approaches of treating HCC. However, recurrence and metastasis were major obstacles for further prolonging of survival after resection. Therefore, many studies aimed to explore new effective drugs to decrease cancer metastasis and relapse, as well as to relieve symptoms. Accordingly, some drugs which have a high specificity and efficiency and low toxicity to patients were produced by chemical synthesis in a current. However, the high cost and time consumption have restricted the development of chemical drugs. In recent years, lots of studies on plants and their derivatives which aim at tumor therapy were prevailing.

*Euphorbia helioscopia *L. is herbaceous plant that is widely distributed in most parts of China. It belongs to the plant family Euphorbiaceae and genus* Euphorbia* [8]. The stem of* E. helioscopia *L. contains a milky liquid that can produce a toxic reaction in the skin and mucous membranes [9]. As a traditional Chinese medicine,* E. helioscopia *L. has been widely used to treat different disease conditions, such as ascites, tuberculosis, dysentery, scabies, lung cancer, cervical carcinoma, and esophageal cancer, for centuries [10–12]. In a word,* E. helioscopia *L. has features of slightly toxic and widely pharmacological effects, no obvious toxic effect when patient takes orally its water decoction 150 g/day clinically, and also no death to mice to fill the stomach with 125 g/kg. To date, numerous studies revealed that the secondary metabolites of* E. helioscopia *L. included diterpenoids [13–20], flavonoids [21, 22], triterpenoids [23], polyphenols [24], steroids, and lipids [17]. In addition, a high content of Quercetin, a plant-derived flavonoid, has been detected in the leaves of* E. helioscopia *L. [25], which have been confirmed to have anticancer properties [26, 27].

More recently, our study found that the extracts of* E. helioscopia *L. had effectively inhibited the growth of human HCC lines SMMC-7721, BEL-7402, HepG2, gastric carcinoma cell line SGC-7901, and colorectal cancer cell line SW-480. After extracts of* E. helioscopia *L., we found EAE had markedly inhibited the proliferation of SMMC-7721 cells in a time and dose dependent manner. EAE treatment arrested cell cycle in G-1 phase and EAE used at the concentration range of 100–200 *μ*g/mL induced a marked increase of subdiploid peak. After EAE treatment at the concentrations of 150 and 200 *μ*g/mL, the percentage of apoptotic cells was increased. At the EAE concentration of 200 *μ*g/mL, the typical morphology of early apoptotic change was observed in SMMC-7721 cells. Inhibited the proliferation of SMMC-7721 cells rely on time and dose shown that the EAE was an active fraction of antitumor [28].

In the present study, we utilized the EAE to treat nude mice xenografts of human HCC and investigated its effect on tumor progression with regard to growth, apoptosis, invasion, and metastasis.

## 2. Materials and Methods

### 2.1. Herbs and Extraction

An entire plant of* E. helioscopia *L. was collected in June 2012 from Dingxi, Gansu, China. The field of collection was approved by the Agriculture Committee of Dingxi, where it did not involve endangered and protected species, and was identified in the Institute of Botany, School of Life Sciences, Lanzhou University and Gansu Institute for Drug Control. The extraction of* E. helioscopia *L. was performed in the Pharmacy of Xuan Wu Hospital Capital Medical University, Beijing, China. The dried* E. helioscopia *L. was ground and extracted with 70% ethanol for 5 hr and then filtered. The filtrate was concentrated by rotary evaporator (BUCHI, Switzerland). Fractionating the extract of concentration was done by petroleum ether, chloroform, ethyl acetate, and n-butanol, individually. The residue from each fractionation step was used to obtain the subsequent fraction. The extracts from each fractionation step were evaporated to dryness under vacuum.

### 2.2. Cell Culture

The human HCC cell line SMMC-772 was purchased from the Cell Bank of Shanghai Institute of Cell Biology, Chinese Academy of Sciences (Shanghai, China). Cells were cultured in RPMI-1640 medium (Gibco, Grand Island, NY, USA). Culture media were supplemented with 10% fetal bovine serum (FBS; Gibco, Melbourne, Australia) and antibiotics (50 U/mL penicillin and 50 *μ*g/mL streptomycin) and maintained at 37°C in a humidified atmosphere of 5% CO_2_.

### 2.3. *In Vivo *Experiments

Four-week-old nude mice were purchased from Vital River Laboratory Animal Technology Co. Ltd. (Beijing, China). These mice were fed a standard rodent diet and water* ad libitum *in an aseptic laminar flow room with 60%–70% humidity at 25°C. This study was approved by the Ethics Committee of the Second Hospital of Lanzhou University (lzuec 20130011). All animals received humane care and all efforts were made to minimize suffering. One week following the arrival of the nude mice, 100 *μ*L cell solutions (containing 2 × 10^6^ logarithmic growth phase tumor cells) were injected subcutaneously into the animals' necks. The mice were observed daily for diet consumption, bowel function, and mental state, and tumor size was measured every 5 days. The length and width of the tumor were measured with Vernier calipers and calculated using the following formula for tumor volume: length × width^2^ × 0.5. On the 5th day after inoculation, EAE was administered daily, for 30 days. The EAE was mixed with sterile water, at a concentration of 1 mg/mL. The mice were randomly distributed into 5 experimental groups (*n* = 6 per group). The 5 experimental groups were as follows: untreated control group, 5-fluorouracil (Fu) treatment group (10 mg/kg/day), and 3 EAE treatment groups (50 *μ*g/mL, 100 *μ*g/mL, 200 *μ*g/mL, resp.). All mice were sacrificed the day after the last treatment; tumor masses were surgically excised and preserved in liquid nitrogen and fixed in 10% formalin and 2.5% glutaraldehyde, respectively.

### 2.4. Immunohistochemical Analysis

For immunohistochemical staining, 4 *μ*m sections were cut from formalin-fixed paraffin-embedded xenografts using a microtome, placed on a flotation water bath at 45°C, placed onto glass slides, deparaffinized in xylene, rehydrated in decreasing concentration washes of ethanol, and rinsed in phosphate buffered saline. Antigen retrieval was performed by incubating the tissue sections in a microwave oven at medium power for 10 minutes with 10 mM citrate buffer (pH = 6.0). The activity of endogenous peroxidase was blocking with 3% hydrogen peroxide in deionized water for 10 minutes and blocking nonspecific binding site of the primary antibody with normal serum for 15 minutes, target protein localization with the first antibody and visualization and color reaction with secondary antibody as described above.

Primary antibodies included caspase-3, bcl-2, bax, CyclinD1, MMP-9, and nm23-H1 rabbit anti-human polyclonal antibody (Santa Cruz Biotechnology Inc., Santa Cruz, CA, USA). The secondary antibody was goat anti-rabbit antibody.

Immunostaining results were reviewed and scored using a light microscope by two pathologists blinded to the treatment group. Positivity of the stained paraffin sections was defined by staining intensity and percentage of tumor cells; the staining intensity of caspase-3, bcl-2, bax, CyclinD1, MMP-9, and nm23-H1 expression was classified semiquantitatively into negative, weak, moderate, and strongly positive (0, +, ++, and +++), respectively.

### 2.5. Scanning Electron Microscopy

Tissue blocks were fixed in 2.5% glutaraldehyde in 0.1 M phosphate buffered saline (PBS, pH 7.2). Tissue blocks were dehydrated using a graded series of acetone and propylene oxide and embedded in epoxy resin Epon-812. Embedded tissues were sliced into ultrathin sections and stained with uranyl acetate and lead citrate. The sections were examined by scanning electron microscope (JEM-1230, Japan).

### 2.6. Western Blotting

For Western blotting analysis, frozen tissue specimens were homogenized in ice-cold lysis buffer containing inhibitors of proteases (Roche Applied Science, Mannheim, Germany). Protein concentration was determined using the BCA protein assay reagent (Pierce, Rockford, IL, USA). Total protein extracts (20 *μ*g/lane) were separated in 10% SDS-PAGE and transferred onto PVDF membranes (Immobilon-P, Millipore, Billerica, MA, USA). The membranes were blocked in 5% skimmed milk powder for 2 hours. To detect target protein, the membrane was incubated with primary antibody and diluted to 1 : 1000 in blocking buffer for 2 hours and then incubated at 4°C for 12 hours. The primary rabbit anti-human polyclonal antibodies were as follows: caspase-3, bcl-2, bax, CyclinD1, MMP-9, and nm23-H (Santa Cruz Biotechnology Inc., Santa Cruz, CA, USA). The secondary antibody, goat anti-rabbit horseradish peroxidase (HRP) (Sigma), was diluted to 1 : 8000 in blocking buffer for 2 hours. As a loading control, the membrane was probed with anti-actin antibody (Sigma). Following treatment, the membrane was washed and developed by enhanced chemiluminescence using an ECL kit (Amersham Pharmacia Biotech).

### 2.7. Statistical Analysis

Statistical analysis was performed with Graphpad Prism software. Statistical differences between groups were determined using Student's *t*-test. A *P* value of ≤0.05 was considered to be statistically significant.

## 3. Result

### 3.1. EAE Inhibits Xenografts Growth* In Vivo*

**Figure 1:**
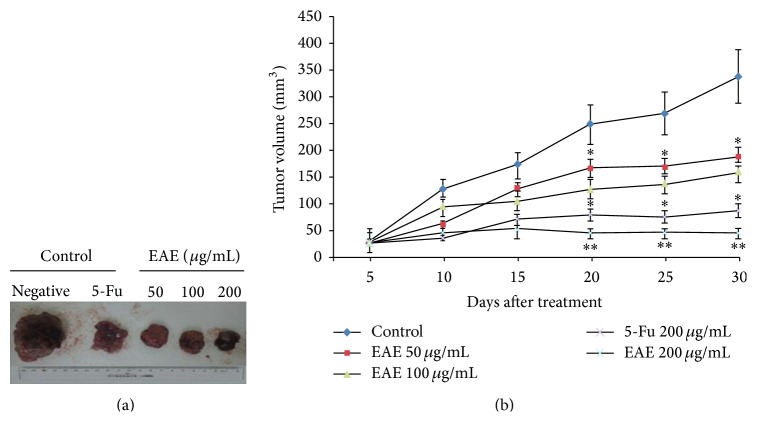
EAE suppresses tumor growth* in vivo*. SMMC-7721 cells (2 × 10^6^) were inoculated subcutaneously into the neck region of nude mice (the volume of xenografts was measured once every 5 days) and treated with EAE. (a) The specimen of xenografts from nude mouse. (b) The growth curve of xenografts. The data represent mean ± S.D of xenografts volume and presented a statistical difference (^∗∗^ *P* < 0.01, ^∗^ *P* < 0.05). In EAE treatment groups, 200 *μ*g/mL significantly inhibited tumor growth compared to the control group (^∗∗^ *P* < 0.01) and better than 5-Fu 200 *μ*g/mL (^∗^ *P* < 0.05), EAE 50 *μ*g/mL (^∗∗^ *P* < 0.01), and 100 *μ*g/mL (^∗^ *P* < 0.05), respectively.

The volume of xenografts was measured in the 5th day after tumor cells inoculation and treatment with EAE by intraperitoneal administration. The tumor growth was inhibited compared to the control group (^∗∗^ *P* < 0.01); with EAE concentration increasing, the tumor volume presented a significant decrease. Compared to control group, the tumor volume was significantly decreased in EAE treatment groups and showed an obvious concentration tendency. The most obvious effect of EAE was 200 *μ*g/mL compared to 50 *μ*g/mL (^∗∗^ *P* < 0.01), 100 *μ*g/mL (^∗^ *P* < 0.05), and 5-Fu 200 *μ*g/mL (^∗^ *P* < 0.05), respectively (Figure 1).

### 3.2. EAE Modulates the Cell Cycle* In Vivo*

To confirm the effect of EAE on the cell cycle* in vivo*, we had detected CyclinD1 protein expression in xenografts and found that it was significantly reduced in the cytoplasm after EAE treatment, compared with the control groups, as measured by immunohistochemical staining (red arrow; Figure 3(a)). After EAE treatment, the protein expression of CyclinD1 was significantly decreased in the EAE treatment groups compared to controls (^∗^ *P* < 0.05, ^∗∗^ *P* < 0.01) detected by Western blot and varied with rising concentration (Figure 3(b)).

### 3.3. EAE Induces Tumor Apoptosis* In Vivo*

**Figure 2:**
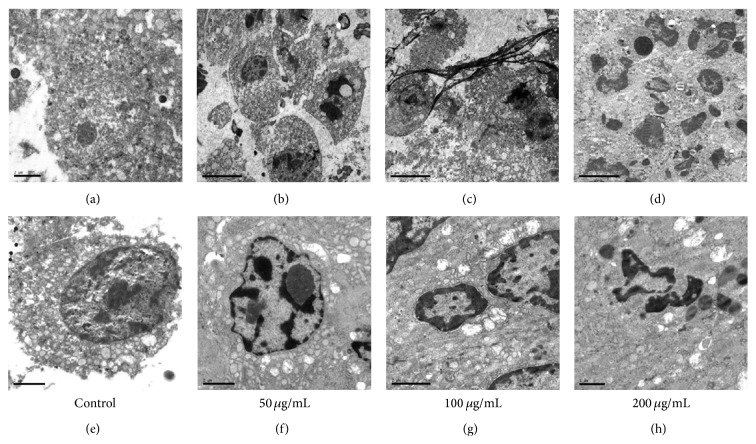
Scanning electron microscope. The ultrastructural morphology of xenografts. ((a) and (e)) Control group. ((b)–(d) and (f)–(h)) 50, 100, and 200 *μ*g/mL EAE treatment groups. The change of ultrastructure in xenografts by treatment with EAE is shown as observed by scanning electron microscopy ((a)–(d), ×6000; (e)–(h), ×12000).

To evaluate EAE induced apoptosis* in vivo*, we observed the ultrastructural morphology of xenografts by scanning electron microscopy and found that the changes of nuclear pyknosis (Figure 2(b)), chromatin condensation, chromatin marginalization (Figures 2(g) and 2(h)), organelle swelling, cytoplasm vacuolization, apoptotic bodies (Figure 2(f)), and fibroplasia surrounded the tumor cells (Figure 2(c)) in EAE treatment groups. Compared with control groups, the expression of bcl-2 (Figures 4(a) and 4(d)) was reduced and that of bax (Figures 4(b) and 4(e)) and caspase-3 (Figures 4(c) and 4(f)) was increased (red arrow) in the cytoplasm after treatment with EAE, showed by immunohistochemical staining.

The result of Western blotting showed that bcl-2 protein expression decreased after EAE treatment; in 200 *μ*g/mL treatment group a significant decrease had appeared compared with control group (^∗∗^ *P* < 0.01) and 5-Fu 200 *μ*g/mL treatment group (^∗^ *P* < 0.05). The protein expressions of bax and caspase-3 were increased after treatment by EAE compared with control group (^∗∗^ *P* < 0.01) and 5-Fu 200 *μ*g/mL treatment group (^∗^ *P* < 0.05; Figures 4(g) and 4(h)).

### 3.4. EAE Suppresses Tumor Invasion and Metastasis* In Vivo*

To determine EAE suppression of tumor invasion and metastasis* in vivo*, MMP-9 and nm23-H1 expressions were examined. Immunohistochemical staining showed that in EAE treatment groups nm23-H1 (Figures 5(b) and 5(d)) expression was increased and MMP-9 (Figures 5(a) and 5(c)) expression was decreased compared to controls (red arrow). By Western blotting, we found that nm23-H1 and MMP-9 protein expression have presented an obvious concentration tendency, compared to controls; nm23-H1 was increased and MMP-9 was reduced, especially in 200 *μ*g/mL group (^∗∗^ *P* < 0.01; Figures 5(e) and 5(f)).

## 4. Discussion

**Figure 3:**
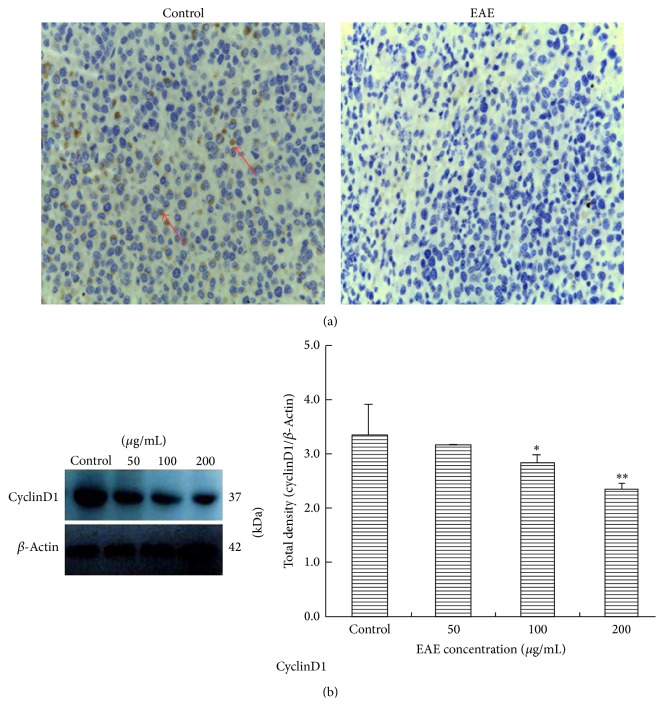
CyclinD1 protein expression in nude mouse xenografts. (a) Immunohistochemical staining detected CyclinD1 in xenografts. EAE treatment group hardly expressed CyclinD1 protein compared to control groups (red arrow). ×400 magnification. (b) Analysis of CyclinD1 protein expression in xenografts by Western blot. Total protein was extracted from xenografts and subjected to Western blot analyses. CyclinD1 protein expression was obviously reduced in EAE treatment groups compared with the control group (^∗^ *P* < 0.05), especially in 200 *μ*g/mL group (^∗∗^ *P* < 0.01), different significantly. *β*-Actin was used as the loading control.

In this study, we assessed the anticancer effect of EAE* in vivo* by the nude mice xenograft model of HCC. The growth was inhibited and the volume significantly decreased after being subjected to EAE treatment for xenografts and shown in dose dependent manner among 50–200 *μ*g/mL EAE compared to control groups (Figure 1). With EAE concentration increasing, the inhibition effect on tumor growth was enhanced. Cell cycle regulation protein CyclinD1 plays a very important role in the G1 phase; the changes of expression were observed in EAE treatment groups (Figure 3). We observed a significant downregulation of cyclinD1 protein expression after treatment with EAE (Figure 3(b)), and the staining in the cytoplasm was reduced compared with controls (Figure 3(a)). Cell cycle data showed that EAE primarily arrested cells in the G-1 phase in a dose and time dependent manner and reduced the percentage of cells in the S phase [28] and that cyclinD1 expression was markedly downregulated. The effect of growth inhibition is mainly mediated by inhibition of cell proliferation, which is associated with a profound modulation of the expression of cell cycle mediators, and the cell cycle machinery disruption; the expression of cyclinD1 was almost completely abrogated with EAE treatment. EAE treatment arrested the cell cycle in the G-1 phase and induced a marked increase of subdiploid peaking. After EAE treatment the percentage of apoptotic cells was increased. The typical morphology of early apoptotic change was observed in SMMC-7721 cells. In addition, EAE treatment displayed a dose dependent inhibitory effect on tumor cell invasion* in vitro *[28].

**Figure 4:**
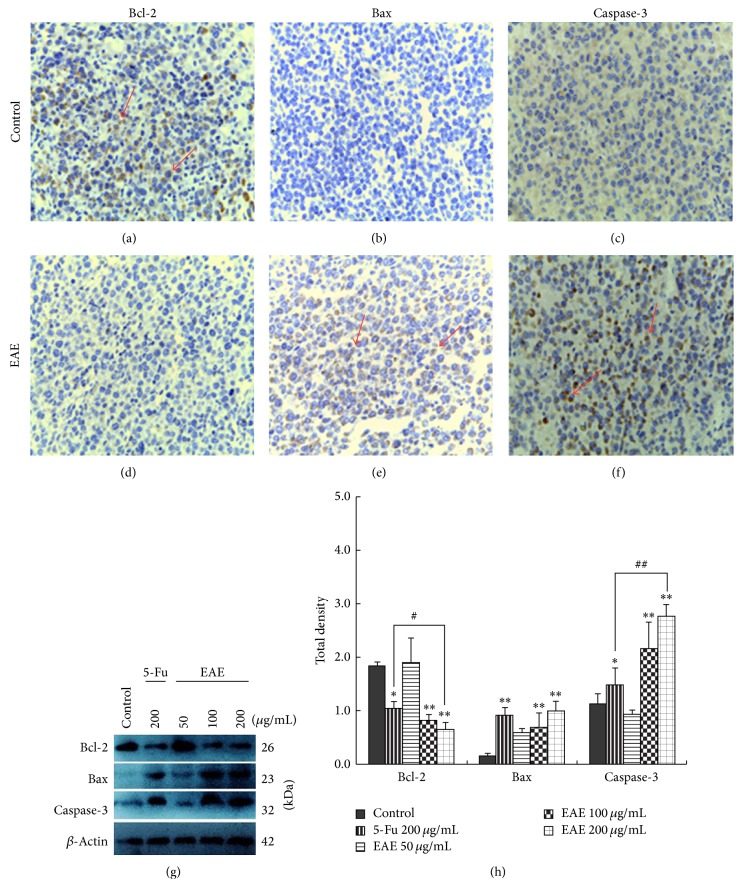
Bcl-2, bax, and caspase-3 protein expressions in nude mouse xenografts. ((a)–(f)) Immunohistochemical staining detected bcl-2, bax, and caspase-3 protein expressions in xenografts. The significant changes of bcl-2, bax, and caspase-3 protein expressions in EAE treatment groups compared to controls; after EAE treatment bcl-2 protein expression was decreased as shown in (a) and (d), but bax and caspase-3 expressions were increased as shown from (b) to (f) (red arrow), ×400 magnification. ((g), (h)) The protein expressions of bcl-2, bax, and caspase-3 in xenografts were analyzed by Western blot. Total protein was extracted from xenografts and subjected to Western blot analyses. As shown compared with the control group (^∗∗^ *P* < 0.01), bcl-2 protein expression was obviously reduced, but bax and caspase-3 expressions were increased. As EAE concentration increasing the quantity of protein expressions have presented a significantly change trend, and the effect of EAE 200 *μ*g/mL better than 5-Fu 200 *μ*g/mL (^∗^ *P* < 0.05).

To induce apoptosis is contributed to EAE inhibiting tumor growth. The Bcl-2 family played a critical role in apoptosis and the members are classified antiapoptosis factors, which mainly include Bcl-2, Bcl-XL, Bcl-W, and proapoptotic factors, which mainly include Bax, Bak, Bik, and Bid. A very important mediating role in apoptosis was caspase family, and caspase-3 is a key effector and functions in many apoptosis signaling transduction pathways [29]. We found that the expression protein of bcl-2 exhibited an obvious declining tendency after EAE treatment, and the bax and caspase-3 exhibited an obvious increasing tendency (Figure 4). This result suggests that EAE, mediating the protein expression of bcl-2 downregulation and bax and caspase-3 upregulation in tumor cells, led to antiapoptosis factor being reduced and proapoptosis factor being increased, to induce cells apoptosis in xenografts. In the EAE treatment groups, the protein expression of bcl-2 was significantly lower than in the control group (*P* < 0.05), and those of bax and caspase-3 were significantly higher than in the control group (*P* < 0.05; Figures 4(g) and 4(h)). Compared with 5-Fu 200 *μ*g/mL group, 200 *μ*g/mL EAE induces apoptosis better than 200 *μ*g/mL 5-Fu, but 50 *μ*g/mL and 200 *μ*g/mL EAE less than 5-Fu 200 *μ*g/mL. As can be seen from these results, EAE can induce apoptosis of HCC* in vivo*, the effect is to enhance with dose increase and better than 200 *μ*g/mL 5-Fu. This demonstrates that EAE exhibits antitumor activity in a dose dependent manner.

**Figure 5:**
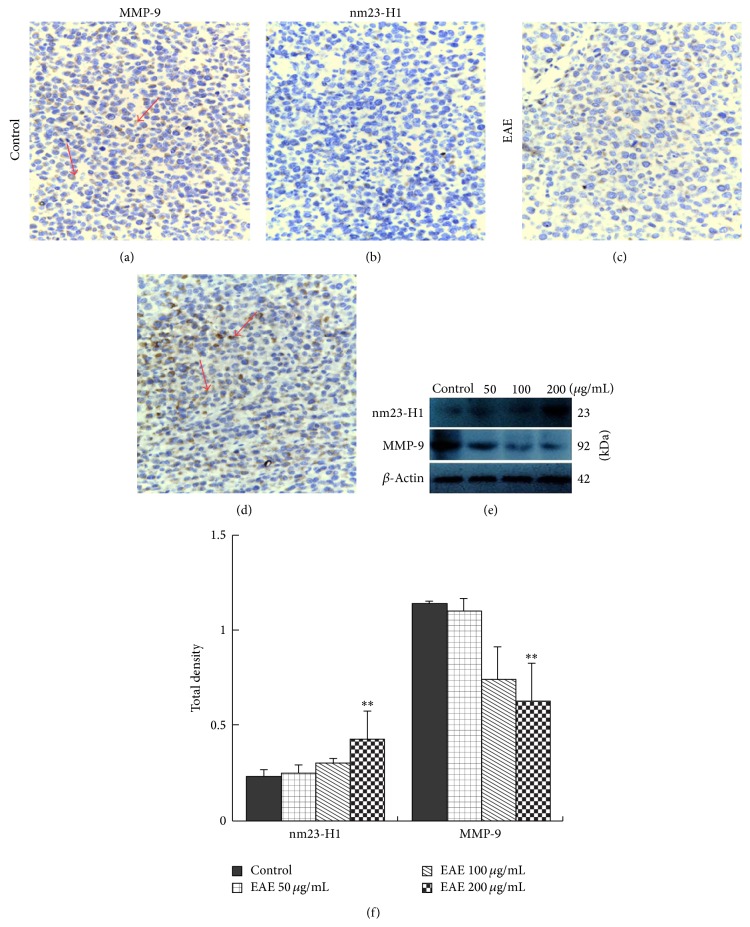
MMP-9 and nm23-H1 protein expressions in nude mouse xenografts. ((a)–(d)) Immunohistochemical staining detected nm23-H1 and MMP-9 protein expressions in xenografts. Compared to control groups, nm23-H1 expression was increased and MMP-9 expression was decreased in EAE treatment groups, as shown ((a) and (c), (b) and (d)) (red arrow). ×400 magnification. ((e), (f)) Western blot analyzed nm23-H1 and MMP-9 protein expressions in xenografts. Total protein was extracted from HCC xenografts and subjected to Western blot analyses for nm23-H1 and MMP-9. Compared with control groups, in EAE treatment groups nm23-H1 protein expression was obviously increased and MMP-9 expression was reduced, especially in 200 *μ*g/mL group (^∗∗^ *P* < 0.01).

Degradation of extracellular matrix by matrix metalloproteinases (MMPs) was a first step for cancer cell migration and invasion, so MMP-9 plays an important role in tumor invasion [30, 31]. The first metastasis suppressor gene, nm23, was identified in 1988 by differential colony hybridization [32]. Nm23-H1 has a variety of validated molecular activities, with at least some playing important roles in regulating its ability to inhibit metastasis [33]. Nm23-H1 and abnormal wing discs, the Drosophila ortholog of Nm23-H1 and Nm23-H2, have also been proposed to be activators of the GTPase dynamin that facilitate endocytosis of growth factor receptors, thereby attenuating their signaling [34–36]. Nm23-H1 and nm23-H2 also interact with proteins involved in cell movement and adhesion [37, 38]. In this study, we found that the protein expression of MMP-9 significantly decreased in EAE treatment groups, and nm23-H1 was significantly increased, compared with the control groups (Figure 5). The change tendency of nm23-H1 and MMP-9 presented in dose dependent manner (Figures 5(e) and 5(f)). Thus, we think that the EAE may have downregulated MMP-9 protein expression and upregulated nm-23H1 in HCC* in vivo*; the more reduced the MMP-9 protein expression the less degraded the extracellular matrix; cancer cell migration and invasion were suppressed, the same effect as nm23-H1 increased in HCC. Although, the development of agents with potential antimetastatic properties is difficult issue, on the basis of these data, we believe that EAE as a new drugs target to play an important role in suppressing tumor metastasis.

## 5. Conclusions

In conclusion, HCC xenografts presented growth inhibition and CyclinD1 protein expression significantly decreased in the G1-phase when subjected to treatment by EAE. To induce cell apoptosis by changing Bcl-2, Bax, and caspase-3 protein expressions in xenografts, the changes of MMP-9 and nm23-H1 protein expressions have shown that the invasion and metastasis of tumor cells may be suppressed by EAE. In sum, EAE can effectively inhibit tumor cells growth both* in vitro *and* in vivo*, making it as an attractive drug candidate. The mechanism of the anticancer effect of EAE remains to be determined. Further research may demonstrate a clinical target for this drug.
